# Sustainable Wood Nanotechnologies for Wood Composites Processed by In-Situ Polymerization

**DOI:** 10.3389/fchem.2021.682883

**Published:** 2021-07-01

**Authors:** Céline Montanari, Peter Olsén, Lars A. Berglund

**Affiliations:** Department of Fibre and Polymer Technology, Wallenberg Wood Science Center, KTH Royal Institute of Technology, Stockholm, Sweden

**Keywords:** wood nanotechnology, biocomposite, nanocellulose, nanostructure, building material, biopolymer, nanocomposite

## Abstract

The development of large, multifunctional structures from sustainable wood nanomaterials is challenging. The need to improve mechanical performance, reduce moisture sensitivity, and add new functionalities, provides motivation for nanostructural tailoring. Although existing wood composites are commercially successful, materials development has not targeted nano-structural control of the wood cell wall, which could extend the property range. For sustainable development, non-toxic reactants, green chemistry and processing, lowered cumulative energy requirements, and lowered CO_2_-emissions are important targets. Here, modified wood substrates in the form of veneer are suggested as nanomaterial components for large, load-bearing structures. Examples include polymerization of bio-based monomers inside the cell wall, green chemistry wood modification, and addition of functional inorganic nanoparticles inside the cell wall. The perspective aims to describe bio-based polymers and green processing concepts for this purpose, along with wood nanoscience challenges.

## Introduction

Bio-based materials are essential for a future sustainable society. With about three trillion trees on earth, wood is one of the most abundant renewable and sustainable material (Crowther et al., 2015). The eco-friendly aspect of sustainably sourced wood is related to its biological origin. The living tree is a renewable resource, and uses solar energy to manufacture the material and also store atmospheric carbon dioxide (CO_2_) in the process. Wood materials with long service life are therefore important for sustainable development through CO_2_ storage. In addition, wooden structures are excellent structural building materials, which can last for thousands of years (Yokoyama et al., 2009).

In pre-industrial age, timber was a key resource for fuel and construction. Despite good mechanical performance of wood structures, the construction of wooden buildings has in this perspective declined in the time frame after the 19th century, because of moisture, degradation and dimensional stability problems, quality variations and flammability challenges compared with alternative materials. Recently, however, engineered wood is revolutionizing the building sector, contributing toward sustainable development; the best example is tall wooden buildings in cities (Cornwall, 2016). Wood has much smaller carbon footprint than steel and concrete, and increased use may reduce CO_2_ emissions by 14% (Oliver et al., 2014).

The porosity of wood provides opportunities for new functionalities. It is possible to combine structural performance with functions to improve energy efficiency of buildings, and add specific properties such as magnetic, luminescent, electrical conductivity and hydrophobicity (Berglund and Burgert, 2018). Another important challenge for load-bearing wood biocomposites is to develop truly sustainable biocomposites based entirely on bio-based fibers and polymers, which are competitive with composites from fossil-based plastics. Although plant fibers may substitute synthetic fibers, bio-based polymers generally do not meet the overall properties and processing requirements for composites. In this perspective, aspects of wood nanotechnologies are discussed, as well as the associated nanoscience challenges and the need for sustainable tailoring methods.

## Wood Composites Used in Load-Bearing Structures

Wood combines high mechanical performance with cost and sustainability advantages. The tree is a renewable resource, wood is available in large volumes at low cost, it has existing infrastructure for energy-efficient harvesting and processing, it is lightweight, and biodegradable. Engineered wood products are widely used for infrastructure applications, carrying substantial dynamic and static loads. There is a variety of engineered wood-based products, including solid structural wood products, laminar and structural composites. Solid-sawn timber is used for glued laminated beams (glulam) made by parallel assembly of sawn boards, and cross-laminated timber (CLT) made of layers assembled crosswise. Since the development of CLT in the 1990s, wooden buildings are constructed at increasing heights (Foster and Ramage, 2020). Other wood composites are fabricated from veneer to produce plywood (made by adhesive bonding of veneer lamellae stacked at different angles), and laminated veneer lumber (made of parallel veneers). These materials are generally used as panels or beams, with little geometrical complexity.

Molded, “wood-plastic composites” (WPCs) are prepared by mixing wood with fossil-based thermoplastics that serves as continuous matrix phase (polypropylene, polyethylene, etc.) (Bourmaud et al., 2018), but are insufficient for most major load-bearing infrastructure applications. There are also technologies for molded, formaldehyde-based thermoset wood fiber composites. In general, modulus for molded WPC and thermoset biocomposites rarely exceeds 7–8 GPa, and the tensile strength is typically 20–60 MPa. The fiber content in molded wood composites can be as high as 50–80 wt%, but the motivation for their use is often low cost. The performance of molded wood composites is often hampered by poorly controlled fiber orientation, and low fiber aspect ratio. The wood fibers tend to be weak, since they are mechanically damaged during processing. These factors in combination, result in poor wood fiber reinforcement efficiency. It is common to add maleic anhydride-grafted polypropylene or polyethylene coupling agents to melt-processed composites, to improve fiber dispersion and interfacial shear strength between the matrix and the wood reinforcement (Keener et al., 2004).

## Targets for Sustainable Wood Nanotechnologies

Increased use of existing, load-bearing forest products, including sawn timber, glulam, LVL-beams, plywood and oriented strand boards (OSB), would contribute toward sustainable development when materials from concrete, gypsum and fossil-based plastics are replaced. Here, we discuss new wood modification concepts at the nanoscale, and possibilities for materials development.

In an attempt to define sustainable wood nanotechnology for the present purpose, the term is here limited to materials or components based on cellular wood structures, so that e.g. wood fiber materials and nanocellulosics are excluded, somewhat arbitrarily. *Sustainable wood nanotechnologies are technologies related to wood substrate (e.g., veneer) modification by nanoparticles, and green modification of the wood cell wall nanostructure by chemical or physical means, to extend the range of wood properties.* An important example is the impregnation of the wood cell wall by monomers or polymer precursors, followed by polymerization. Although formaldehyde-based resins and adhesives in use today can impregnate the cell wall (Stoeckel et al., 2013; Jones and Sandberg, 2020), which in principle is a nanotechnology, they are problematic in the context of sustainable development. Formaldehyde emission is a health hazard, a technical difficulty, and problematic for the perception of eco-friendly materials.

Targets for wood nanotechnologies include improved mechanical performance, since this can reduce the amount of material needed. Wood modification needs to be based on green chemistry principles (Anastas, 2007), and the use of bio-based polymers and nanoparticle technology leading to reduced overall environmental impact. Besides addressing the inherent weaknesses of wood, new functionalities such as heat storage, optical transmittance, fire retardancy and luminescence, can be integrated in the load-bearing wood structure so that eco-indicators for the whole building is improved (Li et al., 2018a). One needs to keep in mind, however, that successful wood nanotechnology depends on the nanoscience of processing-nanostructure and structure-property relationships.

In recent years, bio-based and biodegradable polymers have been considered for replacement of fossil-based polymers in load-bearing biocomposites. Furfuryl alcohol is a bio-based resin used as a substitute for phenol formaldehyde resins (Lande et al., 2008), although the black color and toxicity (monomer and catalysts) are problematic. Other bio-based polymers considered for plant fiber composites include thermosets based on vegetable oils (Williams and Wool, 2000; O’Donnell et al., 2004).

## The Life Cycle of Wood Substrate Biocomposites

**FIGURE 1:**
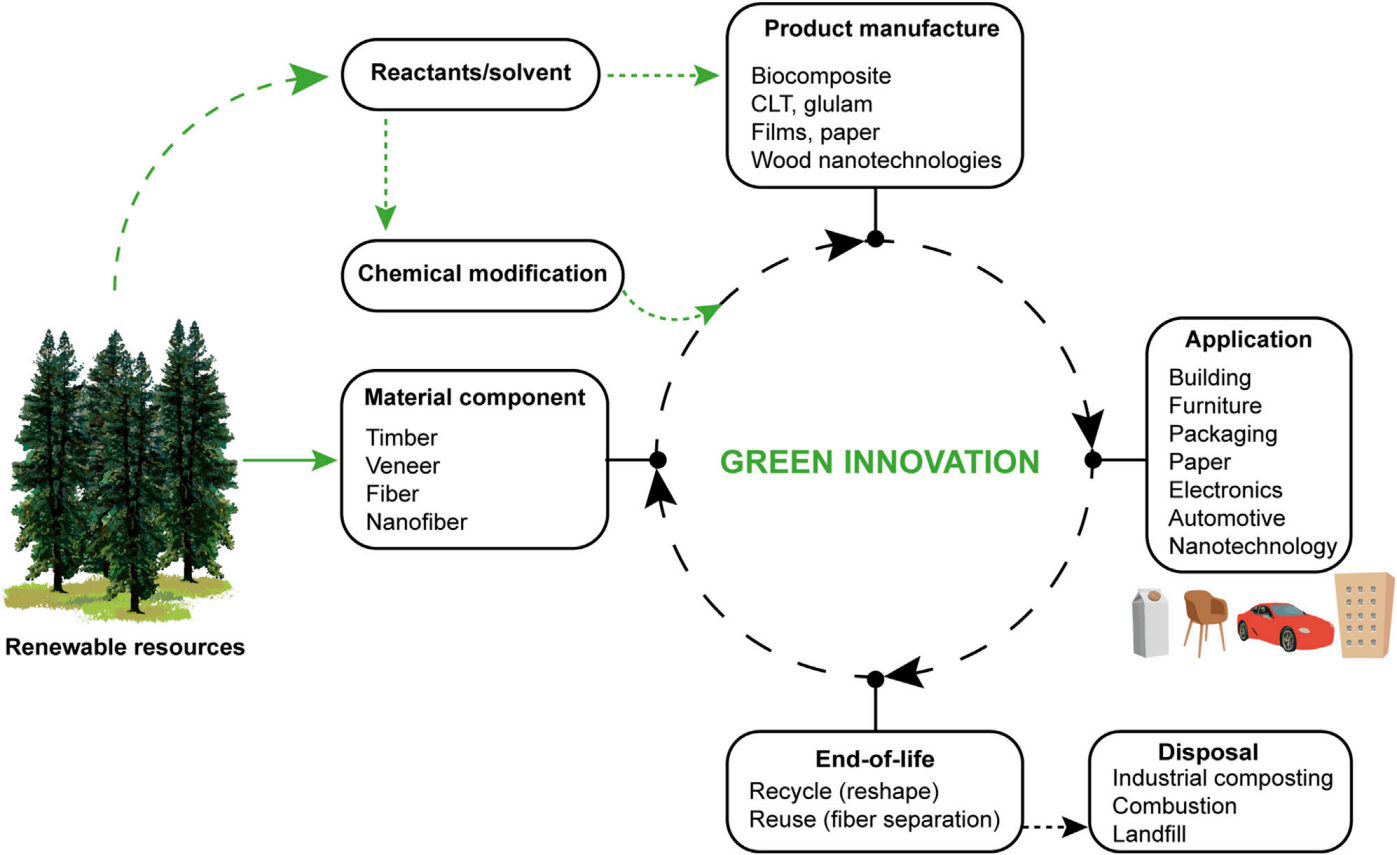
Life cycle for wood-based biocomposites. Sustainable development requires that cumulative energy demand and carbon dioxide emissions are decreased, compared with existing materials.

The present focus is on wood substrate biocomposites where monomers or thermoset precursors are used to impregnate a wood substrate, followed by polymerization. The entire life cycle of the material is illustrated in Figure 1, starting with trees as renewable resources, from which wood-based material components are obtained. They can be modified and processed to form biocomposite products. Note that the composite material is often created during processing of the “product”, e.g., a panel of certain shape. It is used in an application, and after service it can be recycled, reused, upcycled or disposed in different ways. The end-of-life management is dependent on the quality and the possible contaminations of the fiber reinforcement. Sustainable development means economic growth without depletion of natural resources, while retaining ecological balance (Ashby, 2016). Thus, materials from renewable resources is not enough. We need to consider recycling, energy demand from tree to material or product, greenhouse gas emissions, toxicity aspects, and end-of-life management (Ashby, 2013). The CED, cumulative energy demand (sum of energy required during all processing stages), of the product is an important eco-indicator. Low CED is needed in high-performance, sustainable lightweight materials with properties transcending current materials. We need material components from renewable feedstock (Narayan, 2011), green chemistry modification, and low-energy processing.

Lignocellulosic biomass is limited by moisture sensitivity and incompatibility with hydrophobic polymer systems. For wood, the substrate needs modification to address these challenges. Wood contains many different functional groups such as carboxylic acids, aliphatic alcohols, and phenols, useful for this purpose. Often, new chemical treatments are attempted based only on chemical structures of wood biopolymers. This is not sufficient, since the interior parts of the wood substrate, e.g., the wood cell wall, are not readily accessible. Chemical complexity and heterogeneity also means that specific reactions are not straightforward to achieve. The nature of the reaction system is important, and will control both chemical and physical accessibility, for instance by its ability to swell the wood cell wall into a more accessible gel structure.

## Wood Structure and Alternatives for Eco-Friendly Biocomposites

**FIGURE 2:**
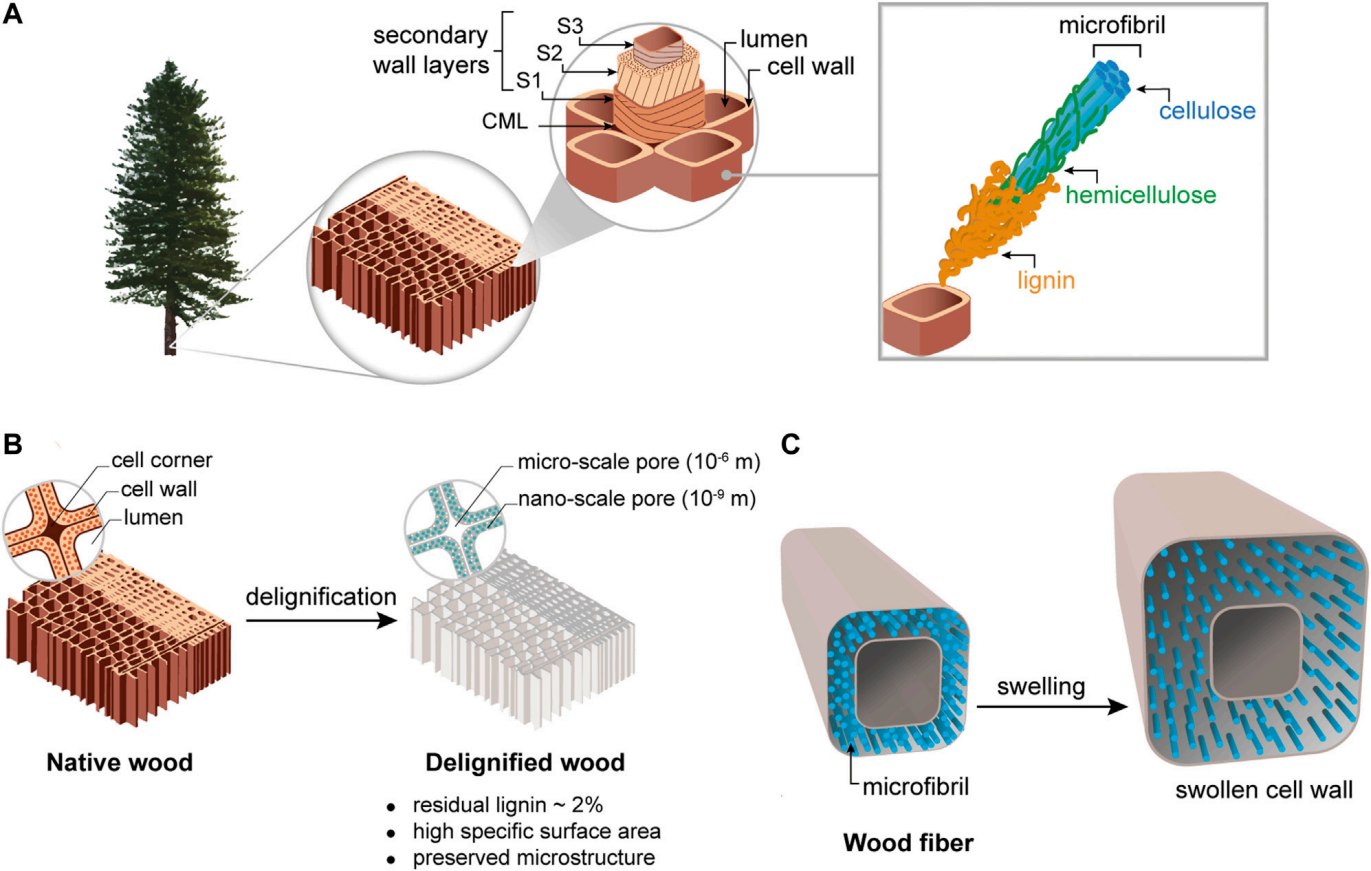
**(A)** Illustration of softwood microstructure and major cell wall components, the starting tissue for nanostructural modification. The cell wall is dominated by the middle lamella, and the thick secondary wall. The cellulose nanofibrils are reinforcing a hydrated mixture of hemicellulose and lignin polymers in the cell wall. **(B)** Wood substrate before and after delignification treatment. Porosity is generated also at nanoscale inside the cell wall. **(C)** Illustration of wood cell wall swelling, which facilitates chemical modification.

Wood is an excellent substrate for load-bearing applications. By filling the pore space in wood with reactants (monomers, thermoset precursors), followed by polymerization, large-scale biocomposites can be prepared for applications in infrastructure. An interesting feature of wood for the sake of modification is its hierarchical structure, which extends from the macroscale to the nanoscale assembly of the cell wall layers and biopolymers (Fratzl and Weinkamer, 2007), see Figure 2. Wood cells are elongated tubular fibers, consisting of a cell wall and a central “lumen” void space. Wood fibers are a few millimeters in length and around 20–50 µm in diameter. The main biological functions of the wood tissue are water transport and mechanical support. In softwoods, fiber tracheids grow thin walls and wide lumen during spring (earlywood) to provide water transport, and thick wall and narrow lumen in the autumn (latewood) for mechanical stability (Figure 2A). In the native tree, the cell wall is itself a nanocomposite with strong, aligned cellulose fibrils embedded in a matrix of lignin, hemicellulose and water. Wood cellulose fibrils consist of axially aligned, extended cellulose chain molecules (Sacui et al., 2014), and have a diameter of 3–4 nm, with an estimated Young’s modulus of 140–200 GPa and a tensile strength of perhaps 7.5 GPa (Nishino et al., 1995; Šturcová et al., 2005; Dufresne, 2017). The fibrils show preferential axial orientation in the cell wall layers, see Figure 2C, and provide strength and stiffness to the cell wall.

The cellulose nanofibrils, CNF, can be disintegrated from chemical wood pulp fibers and used as reinforcement in biocomposites via bottom-up approaches (Kontturi et al., 2018). Disintegration takes place by mechanical means and is facilitated by chemical pretreatment (Pääkko et al., 2007; Saito et al., 2007). Lightweight CNF-based materials can be prepared by a prepreg-approach with potential for semi-structural applications (Ansari and Berglund, 2018). For random-in-plane CNF orientation and 50 vol% CNF, the modulus can approach 10 GPa and the strength exceed 150 MPa. The high energy demand, however, for extracting nanocellulose from the wood pulp fiber cell wall, combined with numerous composite preparation steps (CNF filtering, controlled drying, thermoset precursor impregnation, prepreg stacking and elevated temperature molding), and lack of recycling methods constitute major obstacles to the sustainability of CNF biocomposites. Cumulative energy demand, green-house gas emissions and water depletion indicators are unfavorable for CNF as compared with wood or wood fibers (Oliaei et al., 2021). From a sustainability and processability perspectives, CNF fibrils are problematic for large-scale building materials, but are better suited for films, coatings, aerogels, hydrogels, high-technology devices, or as minor additives in packaging materials.

Wood fibers are generally more eco-friendly than CNF for semi-structural applications, in terms of the eco-indicators mentioned previously. Optically transparent paper and biocomposites are examples of functional materials with good mechanical properties. These materials can be prepared from bleached wood pulp fibers, and offer recyclability potential (Yano et al., 2014; Ansari et al., 2015; Yang and Berglund, 2020b; Yang and Berglund, 2020a). One advantage of wood fiber biocomposites is the possibility to achieve high fiber volume fraction (>50 vol%) for complex, molded geometries by the use of existing processing methods for composites. The main disadvantage is that it is difficult to control fiber orientation in wood fiber biocomposites (Keplinger et al., 2019). If wood substrates are used as the reinforcement, this problem is solved since fibers are highly oriented in the original wood tissue.

## Functionalized Wood Composites With Controlled Nanostructure

Wood substrates, such as veneer, are suitable for sustainable wood composites development. The basic idea is to use the structure of wood as a substrate and reinforcement in wood composites (Berglund and Burgert, 2018), rather than fibers or fibrils. Although fibers and fibrils are suitable for geometrically complex molded composites, wood composites are better for very large load-bearing structures with low eco-indicator values (CED, greenhouse gas emissions, etc.). Top-down modification approaches take advantage of the existing cell wall nanostructure and hierarchical porosity (microscale pore channels at the center of fiber cells, and nanopores inside the cell wall), while preserving the anisotropy of oriented fibers (Chen C. et al., 2020). This porosity provides opportunity for a great variety of functionalization approaches, at different scales. Since the native cell wall is virtually non-porous in the dried state, accessibility is a challenge.

The challenge of cell wall accessibility can be addressed by partial removal of cell wall components. Delignification treatments enable full or partial removal of lignin and hemicelluloses while preserving the oriented wood cellular structure and fibrils (Yano et al., 2001; Frey et al., 2018), see Figure 2B. The remaining lignin content is usually around 1–2% (Keplinger et al., 2020). Hemicelluloses are also affected by delignification and the hemicellulose content is reduced. Delignification generates porosity in the cell wall, and the wood substrate can reach specific surface areas up to 300 g/m^2^ for swollen cell walls, which is favorable for cell wall accessibility (Stamm and Millett, 1941). A swelling agent, such as acetic acid, can expand the cell wall and further facilitate impregnation of molecules or nanoparticles (Figure 2C). Chemical modification effects can then be improved, such as graft density of polymer chains chemically linked to the interior of the cell wall. (Olsén et al., 2020). The delignified cell wall is mesoporous and highly sensitive to drying methods. Drying from water can completely collapse the structure, due to capillary effects. For preservation of porosity and specific surface area, solvent-exchange from water to more non-polar liquids, followed by drying, is a successful approach (Grönquist et al., 2019; Han et al., 2019; Vitas et al., 2019). Although sequential solvent-exchange procedures are helpful (Yamasaki et al., 2019), solvent exchange is not suitable for industrial processing and increases eco-indicator values, but is a valuable tool in nanoscience investigations.

**FIGURE 3:**
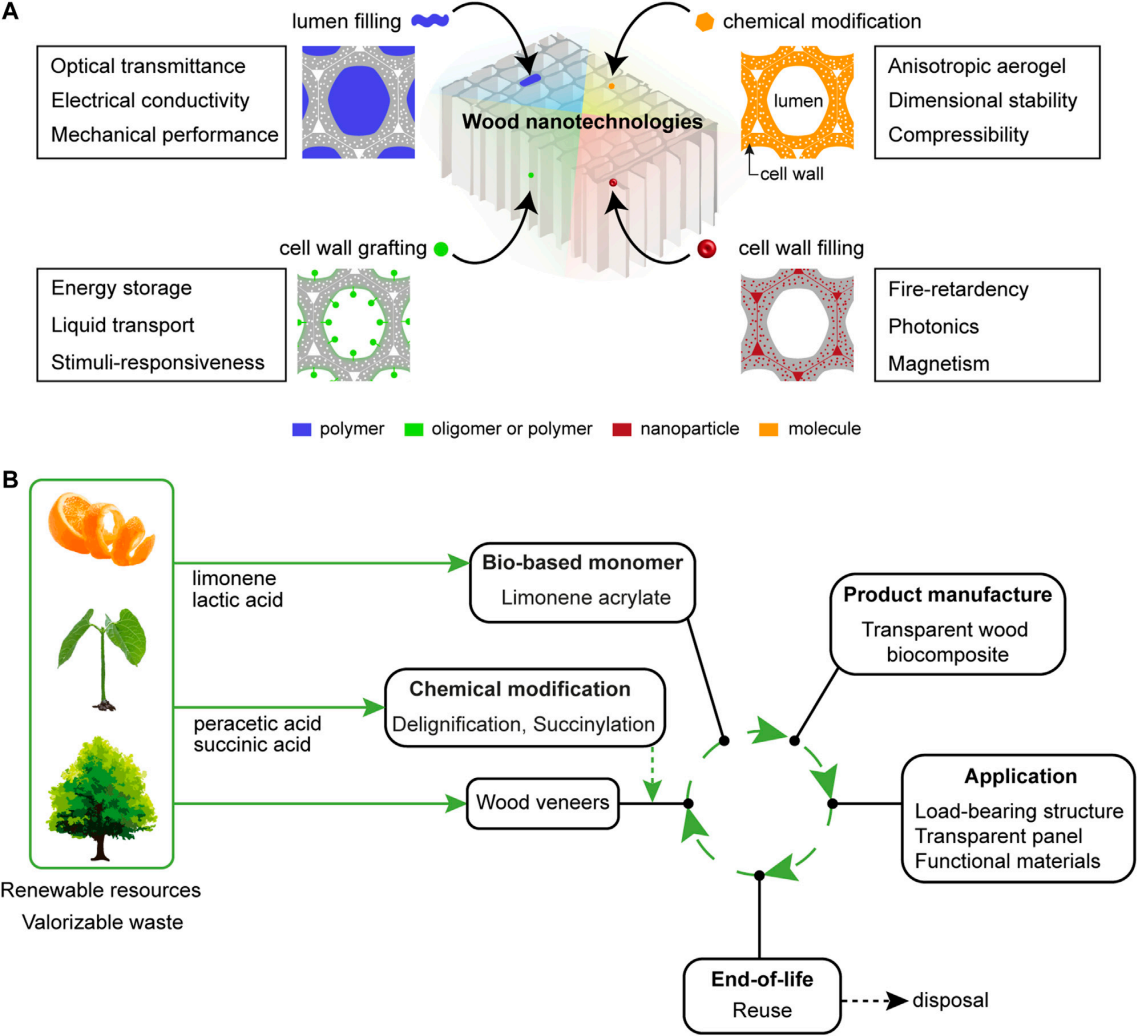
**(A)** Chemical modification strategies for wood substrate functionalization. **(B)** Life cycle of fully bio-based transparent wood biocomposites. The bio-based transparent wood was prepared via green delignification treatment (peracetic acid) followed by solvent-free succinylation (succinic anhydride derived from bio-based succinic acid). A bio-based limonene acrylate monomer, designed from renewable limonene oxide (derived from limonene) and acrylic acid (derived from lactic acid), was impregnated and polymerized inside the wood substrate (Montanari et al., 2021).

The delignified porous wood substrate has poor mechanical properties, because the lignin has been removed, which has an important role for inter-fiber bonding. Filling the lumen with a polymer matrix, see Figure 3A, improves mechanical properties compared with wood (Jungstedt et al., 2020). Densification methods show that strong, anisotropic, homogenous materials can be made from delignified substrates (Yano, 2001; Zhu et al., 2017; Song J. et al., 2018; Frey et al., 2018; Fu et al., 2020; Li et al., 2020). Resin impregnation of the lumen porosity in delignified substrates reduces moisture sensitivity and improves mechanical performance. Frey et al. produced biocomposites with wood content of up to 80 wt%, tensile strength up to 600 MPa and elastic modulus of 70 GPa (Frey et al., 2019). Conducting polymers, metals, and other stimuli-responsive polymer systems have also been successfully infiltrated into lumen space of wood cells to add functionalities (Trey et al., 2012; Keplinger et al., 2016; Wan et al., 2017).

The chemical functionalization philosophy is to target cell wall biopolymers. Hydroxyl groups in particular, can be readily replaced by other functional groups *via* esterification, etherification or graft polymerization. Functionalization can be carried out inside the cell wall, on the inner cell wall surface (lumen-cell wall interface), or in the lumen space (Figure 3A). Cell wall modification has been extensively used to improve dimensional stability and moisture stability. For example, cell wall hydroxyls can be substituted by less polar groups, such as acetyls or even silanes (Donath et al., 2004; Hill, 2006). Acetylation with acetic anhydride is an industrial process, which aims to reduce hydrophilicity of wood by substitution of hydroxyls by acetyl functionalities (Fuchs, 1928; Stamm and Tarkow, 1947; Rowell, 2006). It is a bulking treatment, since the space occupied by water in the native tree is reduced by introduction of acetyls. Another bulking approach relies on the impregnation of monomers or oligomers inside the cell wall (Stamm and Seborg, 1939).

The wood substrate can then be impregnated by monomers, either in the lumen space only or also inside the cell wall (Figure 3A). Various *in-situ* polymerization or grafting polymerization approaches are used to anchor polymer chains within the cell wall (Bach et al., 2005; Cabane et al., 2014; Ermeydan et al., 2014; Burgert et al., 2015; Keplinger et al., 2015). Surface-initiated radical polymerization is a good grafting technique (Cabane et al., 2016), since it is not sensitive to residual moisture. Polymer grafting or cell wall modification can also be used to improve molecular interactions with other polymers in biocomposites (Roy et al., 2009; Carlmark et al., 2012; Herrera et al., 2020). In an unusual cell wall modification procedure, dissolution−regeneration of cellulose nanofibers in lumen space was used to design porous, soft, compressible wood substrates which can serve as aerogels, or compressible materials (Chen et al., 2018; Garemark et al., 2020; Sun et al., 2020).

Wood can be functionalized by nanoparticle impregnation. Inorganic nanoparticles dispersed in water can diffuse inside the nanoporous cell wall for improved wood preservation against microorganisms (*e.g.,* copper carbonate), and against UV degradation (*e.g.,* titanium dioxide) (Evans et al., 2008; Nikolic et al., 2015). Wood mineralization and impregnation approaches using inorganic nanoparticles have also been explored for fire-retardancy applications (Merk et al., 2015; Fu et al., 2017; Zhang et al., 2021). Magnetic particles can be attached on the inner side of the cell wall, at the lumen-cell wall interface, to produce magnetic wood materials for *e.g.,* electromagnetic shielding applications (Merk et al., 2014; Trey et al., 2014; Segmehl et al., 2018). Carbonization strategies followed by nanoparticle infiltration is used to provide electrical properties for energy-storage applications (Chen C. et al., 2017; Luo et al., 2017; Song H. et al., 2018; Wang Y. et al., 2018; Tang et al., 2018; Zhu et al., 2018; Peng et al., 2019; Garemark et al., 2020). Since wood is designed for liquid transport in the tree stem, it can also be functionalized and serve as a membrane for water treatment or oil-water separation purposes (Chen F. et al., 2017; Vidiella del Blanco et al., 2017; Fu et al., 2018a; Guan et al., 2018; Vitas et al., 2018; Ding et al., 2020; Goldhahn et al., 2020; Kim et al., 2020).

## The Case of Transparent Wood

Transparent wood biocomposites were recently developed for structural applications, to combine optical transmittance and structural integrity (Li Y. et al., 2016). Transparent wood is obtained by first removing light-absorbing chemical groups from wood *via* delignification or bleaching treatments (Zhu et al., 2016b; Li et al., 2017a). Then a polymer matrix, commonly acrylates or epoxies, with a refractive index similar to the wood cell wall, is impregnated in monomer form into the porous delignified wood substrate and polymerized to provide optical transmittance. Transparent wood biocomposites are interesting architectural load-bearing structures owing to good mechanical performance, low density (1.2 g/cm^3^), high optical transmittance (≈90%), low thermal conductivity (0.15 W m^−1^ K^−1^), and potential for industrial scaling (Li T. et al., 2016; Li et al., 2018c; Wang X. et al., 2018; Mi et al., 2020). Tensile strength up to ≈270 MPa and elastic modulus of ≈20 GPa could be achieved for transparent wood based on high-density wood species (Jungstedt et al., 2020). The optical properties of transparent wood are attractive because it provides both high transmittance and high haze (≈80%), which is advantageous for diffused lighting and solar cell applications (Zhu et al., 2016a; Li et al., 2017b; Li et al., 2019).

It was recently shown that haze, forward-scattered light, can be tuned by controlling scattering effects within the composite using chemical treatments (e.g., acetylation, bleaching) to improve compatibility at the wood-polymer interfaces (lumen-cell wall and inside the cell wall) or by reducing cellulose content (Li et al., 2018c; Jia et al., 2019; Höglund et al., 2020). Multifunctional transparent wood composites, which combine optical transmittance with other functions, have been reported for applications such as photochromic, electrochromic, heat-shielding, magnetic, and thermal energy storage (Gan et al., 2017a; Lang et al., 2018; Montanari et al., 2019; Qiu et al., 2019; Wang et al., 2019; Samanta et al., 2021). Multifunctional transparent wood composites have been designed by the addition of a functional third-phase component to the polymer matrix and/or the cell wall. For example, quantum dots were added to the polymer matrix to obtain luminescent structures useful in load-bearing lighting applications (Gan et al., 2017b; Li et al., 2017b). The main challenge for multifunctional wood composites is to successfully achieve diffusion of the active component into the cell wall. For instance, nanostructured and multifunctional transparent wood could be prepared by impregnation of a phase-change material inside the cell wall to maximize heat-storage performance (Montanari et al., 2019).

High optical transmittance becomes increasingly difficult as thickness is increased. The reason is that an increased fraction of incoming light is scattered at interfaces between phases of different optical properties. The polymer matrix needs well-matched refractive index to the wood substrate. Chemical treatments can reduce the problem, e.g., by facilitating monomer diffusion into the cell wall so that the defects (nanoscale voids, interface debonds) are minimized (Li et al., 2018b; Chen H. et al., 2019; Chen et al., 2020 H.). To circumvent thickness limitations from processing challenges (incomplete monomer impregnation), laminated plywood structures provide advantages, and can combine high optical transmittance with mechanical performance (Fu et al., 2018b).

## Green Aspects of Wood Functionalization

In a long-term perspective, wood functionalization needs to meet the criteria of green chemistry. The 12 green principles (GPs) are:

Waste prevention **GP-1**.
Atom economy **GP-2**.
Less hazardous chemical synthesis **GP-3**.
Designing safer chemicals **GP-4**.
Safer solvents and auxiliaries design **GP-5**.
Energy efficiency **GP-6**.
Use of renewable feedstock **GP-7**.
Reduce derivatives **GP-8**.
Catalysis **GP-9**.
Design for degradation **GP-10**.
Real-time analysis for pollution prevention **GP-11**.
Inherently safer chemistry for accident prevention **GP-12**.

Wood and cellulose substrates for composites are from renewable resources, but this is not enough (Onwukamike et al., 2019), for sustainable development. Bio-based polymer systems are needed as well as green concepts for composites processing (Curzons et al., 2001). Figure 3B shows the life cycle of a sustainable transparent wood biocomposite, implementing green chemical modification treatments (delignification, succinylation) and impregnation by bio-based monomer. All reactions are carried out without solvent and comply with **GP-1**–**10**.

Biomass as feedstock for chemicals is attractive since CO_2_ becomes an intrinsic part of the biomass/biopolymer structures during biosynthesis. Bio-based polymers and chemicals can serve as building blocks for synthesis of chemicals and polymeric materials (Gallezot, 2007). Waste materials from food, agricultural or forest industries (roots, branches) are particularly interesting feedstock (**GP-7**). The cumulative energy demand for materials and chemicals from biomass waste can be dramatically lower than for petrochemical alternatives. If roots and branches are left in the forest, they will degrade and emit carbon dioxide. If they are burnt, the high moisture content leads to low efficiency, and carbon dioxide emissions. Several bio-based chemicals are industrially produced and commercially available and can be used for large-scale wood functionalization (**GP-7**) (E4tech et al., 2015; Ögmundarson et al., 2020). In the example in Figure 3B, all reactants are from renewable feedstock; green peracetic acid was used for delignification, succinic anhydride from bio-based succinic acid was used for moisture stabilization and compatibilization, while lactic acid and limonene are bio-based building blocks for the limonene acrylate monomer.

Wood modification is motivated by moisture stability, compatibilization with polymers and the opportunity to integrate new functions. Green chemistry means reactants from renewable resources (**GP-7)**, selective modification (**GP-1**, **GP-2**, **GP-8**, and **GP-9**), nonhazardous (**GP-3**, **GP-4**, **GP-5**, and **GP-12**), high atom economy (**GP-2**), mild reaction conditions (**GP-6**, and **GP-9**), energy efficiency (**GP-6**), and at least the same performance as for non-sustainable pathways (Trost, 1991; Anastas and Warner, 1998; Anastas and Eghbali, 2010). Solvents should be replaced by environmentally friendly alternatives (**GP-5**, and **GP-7**), recycled, reduced, or completely removed in order to minimize waste and environmental impact (DeSimone, 2002; Capello et al., 2007; Prat et al., 2013). Here (Figure 3B), a selective and green functionalization using cyclic anhydrides (e.g., succinic anhydride) (**GP-1–5, 6, 7, 10**) was demonstrated under solvent-free conditions for reduced hygroscopicity and facile monomer impregnation (Montanari et al., 2020). This is important, since solvent-assisted monomer impregnation should be avoided in sustainable industrial production of wood composites. The fully bio-based transparent wood biocomposites showed improved optical and mechanical properties, due to high polymer modulus and excellent matching of the refractive index of cellulose (Montanari et al., 2021). Other functionalization strategies have been used to tailor plant fiber properties. For example, green polymer grafting approaches were applied to wood fiber and CNF surfaces through surface-initiated ring-opening polymerization of bio-derived lactones (Lönnberg et al., 2006; Herrera et al., 2020; Olsén et al., 2020). Green delignification treatments for wood substrates were explored such as peracetic acid, ionic liquids, and deep eutectic solvents (**GP-4**, **GP-5**, and **GP-7**) (Miyafuji, 2015; Chen Z. et al., 2019; Montanari et al., 2020). When the purpose of delignification is to remove light-absorbing components, eco-friendly bleaching processes can be employed to remove chromophores while retaining most of the lignin (Li et al., 2017a).

Scientific challenges remain for the development of wood composites with polymers from renewable resources (**GP-7**) (Mohanty et al., 2018). Sustainability of the final product is affected by factors such as feedstock, embodied energy, durability and disposal (Álvarez-Chávez et al., 2012). To be used in high-performance applications, bio-based polymers should offer long-service life for use in structural infrastructures. For scalable technologies, monomers need to be suitable for existing impregnation and polymerization methods for composites processing. Acrylates meet this need, and free radical polymerization is not sensitive to moisture or heterogeneous chemical environments. The bio-based transparent wood biocomposite design in Figure 3B exemplifies the impregnation and polymerization of a bio-derived monomer into a functionalized wood substrate. The limonene acrylate monomer can diffuse into the modified cell wall and polymerizes readily, resulting in high optical transmittance, low haze, and a high strength composite (Montanari et al., 2021). The resulting biocomposite product is a good candidate for sustainable wood nanotechnology.

## Conclusion

Sustainable wood nanotechnologies for wood composites are interesting for infrastructure applications. Wood-polymer composites are structurally efficient by combining high mechanical performance with anisotropy and lightweight. By controlling the composite nanostructure and use lamination to produce large structures, we can extend the property range of wood and add new functions.

Potential wood nanotechnologies are analyzed, where the intrinsic nanostructure is modified. The fact that wood and cellulose are from renewable resources, however, is not enough for wood composites to qualify as truly eco-friendly materials. Excessive energy demands for the processing steps, greenhouse gas emissions, and water depletion effects can be compromising factors. Green chemistry principles are therefore discussed, as guidance for wood composites design and processing. An example of a fully bio-based transparent wood nanocomposite, prepared by a top-down approach, is provided. This improves energy efficiency, since there is no need for nanocellulose disintegration and bottom-up materials preparation. Veneer substrates are instead subjected to green delignification, followed by moisture stabilization by bio-based molecules, impregnation with bio-based monomers in a solvent-free process, and final curing. Optical and mechanical properties are excellent, because of the molecular and nanoscale tailoring. For future work, thermoplastic polymers are desirable, since this would facilitate composites recycling. Future research development should henceforth focus on sustainable tailoring, with systematic implementation of the green chemistry principles and life-cycle assessment with estimations of cumulative energy demand and CO_2_ emissions.

## Data Availability

The original contributions presented in the study are included in the article/Supplementary Material, further inquiries can be directed to the corresponding author.
